# Effect of Polynucleotides on Apex Lingual Regeneration After Amputation Due to a Car Accident: A Case Report

**DOI:** 10.7759/cureus.96990

**Published:** 2025-11-16

**Authors:** Pamela Saavedra, Lee Walker, Paula Arroyo

**Affiliations:** 1 Maxillofacial Regenerative Medicine, Virtus Academy, Santiago, CHL; 2 Dental Surgery, Lee Walker Academy, Liverpool, GBR

**Keywords:** auto-amputation, polydeoxyribonucleotide, regeneration, tongue, ­wound healing

## Abstract

The tongue is an essential organ for swallowing food, speaking, and even breathing. Due to medical reasons or traumatic accidents, the partial loss of this organ can severely affect the quality of life of patients. The tongue has a remarkable capacity for endogenous regeneration. In this case report, the effect of polynucleotides (PDRN) on stimulating tissue regeneration and healing was studied. A relevant recovery in phonemic pronunciation and a reduction in the formation of fibrotic tissue in the scar were observed. These results suggest that PDRN may be a safe and effective adjuvant for promoting natural tongue regeneration.

## Introduction

The tongue is a muscular organ essential for food manipulation and therefore for digestion, taste perception, speech and breathing [1].

Tongue amputation may occur by self-inflicted injuries [2], or maxillofacial trauma with tongue bite [3], or by medical conditions like cancer that need a surgical removal (glossectomy), either partial or total [4].

The partial loss of the tongue causes problems with swallowing, phoneme pronunciation, and taste perception, as well as with the quality of social life (family and work) [5,6].

The tongue is an organ with a significant intrinsic regenerative capacity, observed particularly in partial mutilations. This capacity is attributed to the tissue’s high vascularity and the presence of stem cells that are activated when tissue damage occurs [7].

Despite this tissue repair capacity, the resulting scar can cause stiffness in the natural movement of the tongue [8]. The time of scar healing and the quality of tissue repair are highly influential factors in the patient’s quality of life [9]. 

Polydeoxyribonucleotide (PDRN) has been a promising biomaterial in aesthetic medicine and dermatology. PDRN is composed of a mixture of deoxyribonucleotides derived from salmon sperm. Recent studies suggested that PDRN plays a crucial role in cellular regeneration and tissue repair [10]. The PDRN ameliorated wound healing by enhancing tissue repair through extracellular matrix remodeling, as well as promoting cell growth and angiogenesis [11].

Considering the mechanism of wound healing and the tissue repair characteristics of PDRN that have been mentioned previously, this article aims to present a case report where the effect of PDRN on tongue regeneration and its functional rehabilitation was evaluated.

## Case presentation

A 34-year-old man underwent a partial tongue amputation of 30% after a car accident. He did not have relevant comorbidities. He was received in the Emergency Hospital (Santiago, Chile), where the bleeding was stopped, the tongue was cleaned, and the wound was sutured.

After day 12, the treatment to stimulate tongue regeneration was initiated. Nuclear Healer PN®, containing 25mg/mL polydeoxyribonucleotides and lidocaine 3 mg/mL (Koru Pharma), was injected once a week for four weeks with microinjections of 0.05 mL in the apical region of the tongue up to a volume of 1.5 mL. Strataderm®, a silicone gel, was used (once a day after oral cleaning for four weeks) as a coadjutant therapy to reduce the formation of fibrotic tissue in the wound scar. The follow-up continued once a month up to three months after tongue regeneration treatment. The patient was informed about the procedure before its execution and signed an informed consent.

The functional rehabilitation of the tongue was initiated three weeks after the car accident. The daily exercises were oriented to improve the following dental (/t/, /d/, /n/) and velar (/k/, /g/, /x/) phonemes. As part of the functional rehabilitation, the patient was advised to perform daily oral physiotherapy exercises aimed at improving tongue mobility. These exercises consisted of intensive repetition of dental (/t/, /d/, /n/) and velar (/k/, /g/, /x/) phonemes under speech therapy supervision, over a period of four weeks.

**Figure 1:**
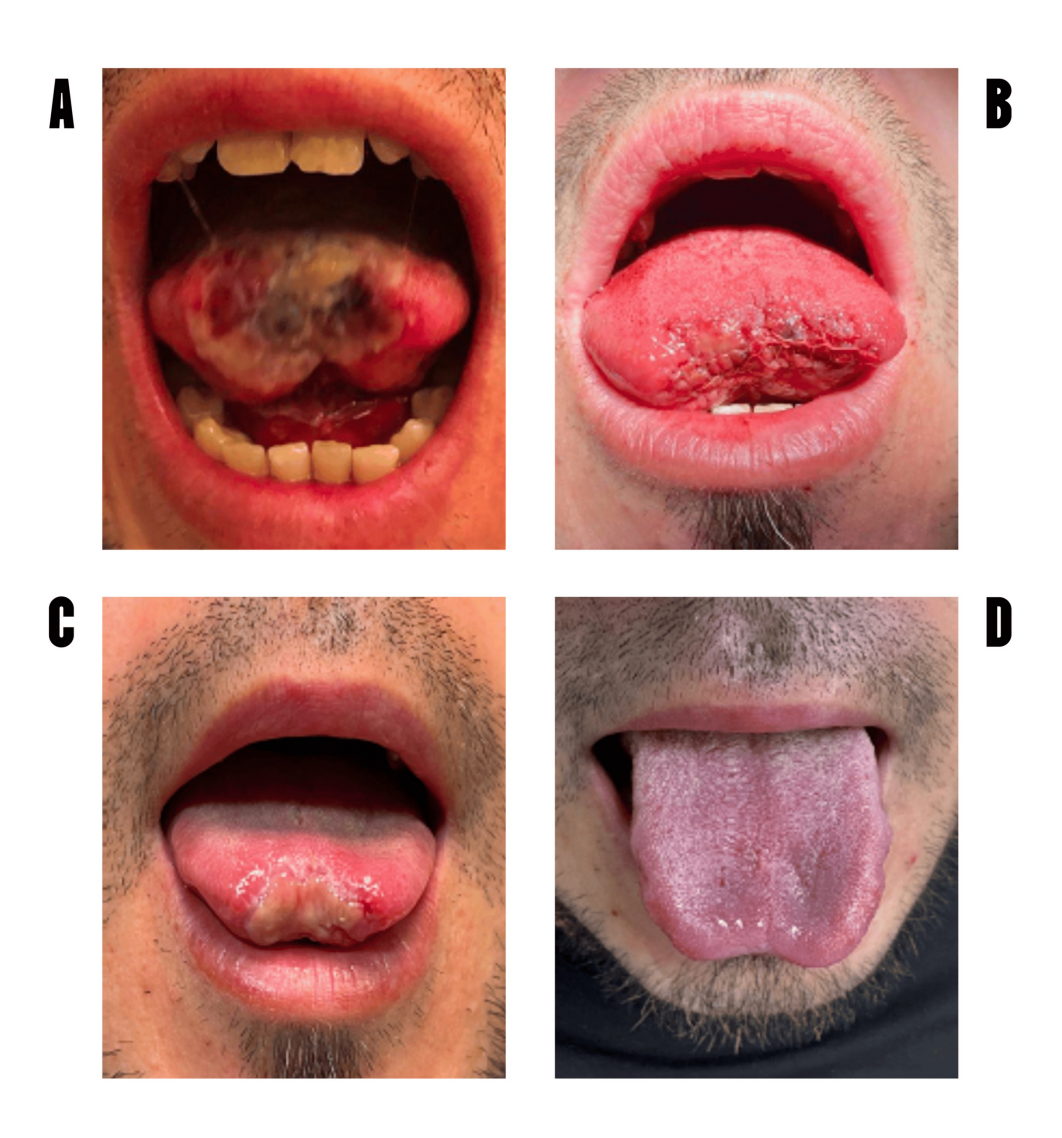
Effect of surgical and pharmacological treatments on tongue regeneration (A) basal tongue traumatism, (B) the tongue aspect after the surgical procedure, (C) the tongue cicatrization at basal condition (12 days after surgery) and (D) at the end of regeneration treatment (eight weeks).

Figure 1 describes the recuperation of volume and wound aspect during the process of tongue rehabilitation.

Functional tongue rehabilitation was determined by: (i) the phonetic evaluation of /t/, /k/, and /n/ showed an increase of 3 points with respect to basal conditions; (2) the Eating Assessment Tool-10 (EAT-10) score was reduced from 14 to 4 points [12], and (3) a quality of life test (SF-36), had an improvement of 25% in mental and 30% in physical aspects [13]. The subject’s testimony was: At the beginning, I couldn’t pronounce the phoneme /k/; the trauma limited my speech a lot. However, week by week, I felt that my tongue mobility was returning. After three months, I speak almost normally. These treatments were very important for him because he is a singer.

## Discussion

The present study is the first evaluation of the safety and efficacy of PDRN in stimulating the regeneration of the tongue and the remodeling process in scar formation.

In recent years, therapeutic strategies based on the use of human mesenchymal stem cells [14], exosomes from human gingiva-derived mesenchymal stem cells [15], and tissue engineering [16] have been evaluated in a preclinical setting to stimulate tongue regeneration with promising results.

Regarding the stimulation of wound healing on the tongue, the use of autologous plasma-rich growth factors was evaluated in an animal model (rabbit), and a favorable effect on epithelium formation and resolution of wound inflammation was observed at 28 days of follow-up [17]. In addition, a preclinical study demonstrated that the use of erythropoietin hydrogel can accelerate regeneration of the tongue by reducing tissue inflammation and enhancing neovascularization in rats [18].

Recently, a systematic review of clinical outcomes about the use of PDRN in the oral cavity, particularly in periodontal regeneration, was conducted. The authors analyzed four clinical trials, concluding that the PDRN seems to provide significant benefits in periodontal regeneration by enhancing bone and tissue healing and reducing inflammatory responses [19].

## Conclusions

The use of PDRN emerged as an effective alternative for skin rejuvenation, which led to its study in skin pathologies or disorders that require additional stimulation for tissue regeneration and repair. These characteristics prompted its use in this case of tongue amputation. These results suggest that PDRN can be an effective adjuvant treatment to promote the natural process of tongue regeneration and wound healing, both physically and functionally. Further studies must be conducted to confirm these results.

## References

[1] Kajee Y, Pelteret JP, Reddy BD (2013). The biomechanics of the human tongue. Int J Numer Method Biomed Eng.

[2] Munerato MC, Moure SP, Machado V, Gomes FG (2011). Self-mutilation of tongue and lip in a patient with simple schizophrenia. Clin Med Res.

[3] Hernández-Méndez JR, Rodríguez-Luna MR, Guarneros-Zárate JE, Vélez-Palafox M (2016). Traumatic partial amputation of the tongue. Case report and literature review. Ann Med Surg (Lond).

[4] Ansarin M, Bruschini R, Navach V (2019). Classification of GLOSSECTOMIES: proposal for tongue cancer resections. Head Neck.

[5] Dzioba A, Aalto D, Papadopoulos-Nydam G (2017). Correction to: functional and quality of life outcomes after partial glossectomy: a multi-institutional longitudinal study of the head and neck research network. J Otolaryngol Head Neck Surg.

[6] Catford JC, Esling JH (2006). Phonetics, articulatory. Encyclopedia of Language & Linguistics.

[7] Ha P, Liu TP, Li C, Zheng Z (2023). Novel strategies for orofacial soft tissue regeneration. Adv Wound Care (New Rochelle).

[8] Bigcas JLM, Okuyemi OT (2025). Glossectomy. StatPearls [Internet].

[9] Stone M, Langguth JM, Woo J, Chen H, Prince JL (2014). Tongue motion patterns in post-glossectomy and typical speakers: a principal components analysis. J Speech Lang Hear Res.

[10] Lee KW, Chan KW, Lee A, Lee CH, Wan J, Wong S, Yi KH (2024). Polynucleotides in aesthetic medicine: a review of current practices and perceived effectiveness. Int J Mol Sci.

[11] Shin J, Park G, Lee J, Bae H (2018). The effect of polydeoxyribonucleotide on chronic non-healing wound of an amputee: a case report. Ann Rehabil Med.

[12] Belafsky PC, Mouadeb DA, Rees CJ, Pryor JC, Postma GN, Allen J, Leonard RJ (2008). Validity and reliability of the Eating Assessment Tool (EAT-10). Ann Otol Rhinol Laryngol.

[13] Ware JE Jr, Sherbourne CD (1992). The MOS 36-item short-form health survey (SF-36). I. Conceptual framework and item selection. Med Care.

[14] Yamashita Y (2007). Poster 153: the experimental study for regeneration of the tongue with human mesenchymal stem cells. J Oral Maxillofac Surg.

[15] Zhang Q, He P, Shi S (2025). Secretome enriched with small extracellular vesicles derived from human gingiva-derived mesenchymal stem cells enhances rat tongue muscle regeneration. J Nanobiotechnology.

[16] MacDonald AF, Gross AJ, Jones BJ, Dhar MS (2022). Muscle regeneration of the tongue: a review of current clinical and regenerative research strategies. Tissue Eng Part B Rev.

[17] Jornet MP, Alonso FC, Miñano FM, Ortega V (2010). Effects of plasma rich in growth factors on tongue wound healing: experimental study in rabbits (Article in Spanish). Medicina Oral Patología Oral y Cirugía Bucal.

[18] Ata F, El-Qashty R, Farid M, Youssef J (2024). Healing of induced tongue defects using erythropoietin hydrogel (an experimental study on rats). BMC Oral Health.

[19] Mari R, Ramamurthy J, Rudhra K, Krishnaswamy N (2025). Efficacy of polydeoxyribonucleic acid (PDRN) in periodontal regeneration: a systematic review of clinical outcomes. J Oral Biol Craniofac Res.

